# Physical activity and sleep behaviour in women carrying BRCA1/2 mutations

**DOI:** 10.1038/s41598-022-16687-1

**Published:** 2022-07-27

**Authors:** Letizia Galasso, Lucia Castelli, Eliana Roveda, Andreina Oliverio, Ivan Baldassari, Fabio Esposito, Antonino Mulè, Angela Montaruli, Patrizia Pasanisi, Eleonora Bruno

**Affiliations:** 1 Department of Biomedical Sciences for Health, University of Milan, Via G. Colombo 71, 20133 Milan, Italy; 2 IRCCS Istituto Ortopedico Galeazzi, Via R. Galeazzi 4, 20161 Milan, Italy; 3 Department of Research, Fondazione IRCCS Istituto Nazionale dei Tumori di Milano, Via G. Venezian 1, 20133 Milan, Italy

**Keywords:** Cancer, Pathogenesis, Risk factors

## Abstract

The aim of the present study is to explore the potential association between sleep quality and physical activity (PA) in women carriers of *BRCA1/2* mutations. 63 women completed the Pittsburgh Sleep Quality Index (PSQI) and Godin Shepard Leisure-Time Physical Activity Questionnaire (GSL-TPAQ) and were included in the present cross-sectional analysis. Globally, women showed a PSQI score of 7.0 ± 3.6 and a GSL-TPAQ score of 22.8 ± 18.3. *Good sleepers* (PSQI score ≤ 5) showed significantly higher PA levels compared to *bad sleepers* (PSQI score > 5)*.* Women in the higher tertile of GSL-TPAQ total score (≥ 27 METs/week) have a prevalence ratio (PR) of being a *good sleeper* of 2.85 (1.25–6.52, 95% confidence intervals) compared to women in the lower tertile (≤ 11 METs/week). These results were consistent in BRCA1 and BRCA2 women. Considering each single question of PA intensity, the PR of being a *good sleeper* by unit of increase of MET/week was higher and significant in women engaged in strenuous and moderate intensity PA. These results suggests a direct association between PA and sleep quality in women carriers of BRCA mutations.

## Introduction

Women carrying *BRCA1/2* mutations have a 55% lifetime risk (penetrance) of developing breast cancer (BC)^1^ and the percentage is even higher at age 80 with 72% (95% CI 65–79%) for *BRCA1* and 69% (95% CI 61–77%) for *BRCA2* mutation carriers^2^. In these women the penetrance for ovarian cancer (OC) is also higher, in the order of 16–59%^1,3^. Epidemiological studies have suggested that the penetrance of *BRCA1/2* genes may be modulated by a number of lifestyle factors. Greater body weight and life-long weight gain have been found associated with an increased BRCA penetrance^4–6^ especially for post-menopausal BC^7,8^ and for OC^9^*.* Consistently, higher fat mass, severe metabolic syndrome (MS) and high serum levels of insulin and insulin-like growth factor-I (IGF-I) affect BRCA penetrance^10^.

In recent years, sleep disorders are emerging environmental factors that influence the risk of BC^11–14^ and are co-morbid with the MS. The association between sleep and BC may be explained by misalignment of the molecular clock circadian rhythm that decreases melatonin secretion, an oncostatic agent and estrogen suppressor^15–17^ and by altering the regulation of glucose tolerance and insulin-sensitivity^18,19^. Sleep problems or bad sleep quality increase after BC diagnosis and treatment and are more common in *BRCA1/2* mutation carriers compared to general population^20^. In fact, sleep disturbances are very frequent post-surgical symptoms (prevalence of 46.7%) in the BRCA carriers who chose risk reducing surgery for primary prevention^21^.

Physical activity (PA), especially during adolescence or early adulthood, affects BC risk in women with *BRCA1/2* mutations^22–24^.

PA improves sleep quality and quantity and reduces obesity and MS parameters^25–28^. PA has a protective effect on sleep in both healthy subjects and cancer patients, including sporadic BC women^26,29,30^. PA is a synchronizer of the circadian rhythms by increasing the activity of sympathetic nervous system that modulates melatonin secretion^31^.

Furthermore, high intensity exercise upregulates the activity of peroxisome proliferator-activated receptor g (PGC-1a) that regulates the expression of the clock gene family, that are involved in sleep behaviour and insulin sensitivity^32^.

Despite the aforementioned evidence, there are no studies on the association of PA and sleep behaviour in women carriers of *BRCA1/2* mutations. In the present paper we aim to explore the potential relationship between sleep quality and PA in a cohort of BRCA women who joined our dietary intervention trial^10,33–37^.

## Materials and methods

### Study design and participants

The design of the Italian multicenter dietary intervention trial on women carriers of *BRCA1/2* mutations (reference number: NCT03066856) and its main results have been already described and reported^10,33–37^. In brief, the trial aimed to test if a 6-month dietary intervention based on the Mediterranean diet (MedDiet) with a moderate protein restriction significantly reduces potential modulators of *BRCA1/2* penetrance such as body weight, IGF-I, insulin and main factors of MS. Eligible subjects were women aged 18–70 years, carriers of *BRCA1/2* mutations, with or without a previous diagnosis of breast and/or ovarian cancer and without clinical evidence of metastases. Unaffected women who underwent bilateral prophylactic mastectomy did not enter the cohort study. All participants signed informed consent, filled questionnaires about their medical history and dietary habits, underwent anthropometric and body composition assessment and donated 20 mL of a blood sample. Anthropometric and body composition measurements, blood samples and dietary data were provided at baseline and the end of the dietary intervention. Among the 502 volunteers randomized into the trial, 63 women recruited between October 2018 and June 2019 also reported at baseline, before starting the dietary intervention, data on sleep and PA habits and were included in the present cross-sectional analysis.

The Ethics Committee of Fondazione IRCCS Istituto Nazionale dei Tumori di Milano approved the study (approval number: INT106/13).

### Sleep and physical activity data collection

Two additional questionnaires for the assessment of sleep and PA habits (Pittsburgh Sleep Quality Index—PSQI and Godin Shepard Leisure-Time Physical Activity Questionnaire—GSL-TPAQ, respectively)^38,39^ were filled by the volunteers included in the present analysis.

The PSQI assesses sleep quality and habits in the previous 30 days. This is a retrospective self-report questionnaire, based on 19 items, in order to evaluate seven sleep components: (i) perceived sleep quality, (ii) sleep latency, (iii) sleep duration, (iv) sleep efficacy, (v) sleep disturbances, (vi) use of sleep medications, (vii) daytime dysfunctions. The score for each question ranges between 0 (no problem at all) and 3 (several problems). The sum of the seven components returns a final score from 0 to 21, with lower scores indicating a better sleep quality. The cut-off value of 5 divides participants into *good* sleepers (0–5) from *bad* sleepers (6–21). In addition, the numbers of hours spent in bed and sleeping and the sleep efficiency can be obtained from the questionnaire^38,40^.

The GSL-TPAQ^39^ assesses the amount of leasure time PA performed in the last seven days by including three questions about frequency of PA (number of activities/week). Each question refers to PA of different intensity (one question on mild, one on moderate and one on strenuous PA) which corresponds to specific Metabolic Equivalent of Task (MET) amount. Mild PA corresponds to 3 METs, moderate PA to 5 METs and Strenuous PA to 9 METs. The total GSL-TPAQ score is obtained using the following formula: (frequency of mild × 3) + (frequency of moderate × 5) + (frequency of strenuous × 9). If the final score is < 24, the subject is classified as *inactive*, while in the opposite case (≥ 24) is described as *active*^39^.

### Statistical analysis

General characteristics of the study population were summarized by *BRCA1/2* using frequencies or means and standard deviation (SD), and compared using χ^2^ or *t* tests, as appropriate.

By using the cut-off of the PSQI score, the study women were stratified into *good* (PSQI score ≤ 5) and *bad* sleepers (PSQI score > 5). Women’s metabolic and anthropometric characteristics were therefore summarized and compared with ANCOVA controlling for age and BMI. Since stratifying the population by disease status (affected and unaffected) or by age (below and above the median value of 48.8 years) we did not find any significant difference for all the variables under study, we decided to perform the analyses in the whole population and by BRCA mutation type.

As regards GSL-TPAQ, we considered both the total score and the results for each question of different PA intensity (strenuous, moderate, mild) separately in all the analyses.

We conducted a correlation analysis (*Spearman* coefficient) among all anthropometric, metabolic, sleep and PA data.

Since the aim of the present study was to describe potential associations between PA and sleep quality, we performed a binomial regression model to estimate the prevalence ratios (PRs) and 95% confidence intervals (CI) of being a *good sleeper* by increasing PA levels (increasing tertiles of GSL-TPAQ total score) in the whole population and in BRCA1 and BRCA2 women, controlling for age and BMI. We also repeated the PR analysis by MET/week increase taking into account each question of PA intensity (strenuous, moderate, mild).

A p-value of < 0.05 was taken as significant. The statistical analyses were carried out using STATA 14 (StataCorp–College Station, TX, USA) and IBM Statistical Package for the Social Sciences—SPSS Statistics version 27 (IBM Corp. Released 2019. IBM SPSS Statistics for Windows, Armonk, NY: IBM Corp).

### Ethics declarations

The study was conducted according to the guidelines of the Declaration of Helsinki, and approved by the Ethics Committee of Fondazione IRCCS Istituto Nazionale dei Tumori di Milano (approval number: INT106/13).

### Consent to participate

Informed consent was obtained from all subjects involved in the study.

## Results

The general characteristics of the study population by *BRCA1* and *BRCA2* are reported in Table 1. 63 women with *BRCA 1/2* mutations (mean age, 47.6 ± 12.5 years), 39 with *BRCA1* and 24 with *BRCA2* mutation, were included in the present investigation. The distribution of general characteristics was homogeneous between BRCA1 and BRCA2 women. Taking the whole population, women presented on average a PSQI score of 7.0 ± 3.6 and a GSL-TPAQ score of 22.9 ± 18.3. As regards sleep quality and PA levels we did not found any significance difference due to the BRCA mutations.

**Table 1:** Baseline characteristics of the study population.

Variables	Total (63)	BRCA 1 (39)	BRCA 2 (24)	*p* value*
**Age (years)**	47.6 ± 12.5	47.4 ± 13.0	47.9 ± 11.8	0.87
**Education (%)**				
First level	22.2	23.1	20.8	
Second level	44.4	41.0	50.0	
Third level	33.3	35.9	29.2	0.78
**Menarche (yrs)**	12.8 ± 1.5	12.8 ± 1.5	12.9 ± 1.5	0.75
**Menopause (%)**	42.8	48.7	33.3	0.23
**Oral contraceptive in the past (%)**	61.9	61.5	62.5	0.94
**Smoke in the past (%)**	14.3	10.3	20.8	0.26
History of a previous cancer (%)	66.7	61.5	75	0.27
**PSQI score**	7.0 ± 3.7	7.1 ± 3.8	6.8 ± 3.5	0.75
**GSL-TPAQ score**	22.9 ± 18.3	22.7 ± 18.6	23.1 ± 18.2	0.94

Frequencies (%), mean ± SD values.

*p < 0.05.

### Comparison between good and bad sleepers

Table 2 shows the baseline anthropometric, metabolic and PA data of the study population stratified by *good* (n = 26, 41, 3%) and *bad sleepers* (n = 37, 58, 7%) in the whole population and in BRCA1 and BRCA2 women. Referring to anthropometric and metabolic parameters, *bad sleepers* showed a slightly worse condition compared to *good sleepers* without any significant result. In the whole population, we observed that *bad sleepers* showed a significantly lower total GSL-TPAQ score. These results were consistent also in BRCA1 and BRCA2 mutation carriers. As regards the single question of different PA intensity (strenuous, moderate, mild), we found that *bad sleepers* showed significantly lower levels of strenuous, moderate and mild PA compared to *good sleepers* (*p* < 0.01; *p* < 0.01; *p* < 0.05, respectively). As regards the stratification by BRCA mutation type we observed consistent results. However, in BRCA1 women the results were significant only for moderate levels of PA while in BRCA2 we observed significant results for strenuous and mild PA.

**Table 2:** Baseline characteristics by *good* and *bad sleepers* in the whole population and in BRCA1 and BRCA2 women.

Variables	TOTAL		BRCA 1		BRCA 2	
	Good sleepers (*n* = 26) Mean ± SD	Bad sleepers (*n* = 37) Mean ± SD	Good sleepers (*n* = 15) Mean ± SD	Bad sleepers (*n* = 24) Mean ± SD	Good sleepers (*n* = 11) Mean ± SD	Bad sleepers (*n* = 13) Mean ± SD
Age (years)	45.0 ± 13.8	49.5 ± 11.3	42.7 ± 14.9	50.4 ± 11.1	48.3 ± 12	47.7 ± 12.1
Weight (kg)	61.1 ± 10	64.9 ± 10	61.1 ± 11.2	64.0 ± 9.7	61.0 ± 8.5	66.5 ± 10.9
BMI (kg/m^2^)	23.5 ± 4	25.1 ± 4.2	23.6 ± 3.9	24.7 ± 4.3	23.2 ± 4.3	25.7 ± 4.1
Waist circumf. (cm)	71.6 ± 9.2	75.5 ± 11	71.4 ± 8.5	74.6 ± 11.5	71.8 ± 10.5	77.2 ± 10.1
Hip circumf. (cm)	95.8 ± 8.1	98.5 ± 8.4	95.9 ± 8.8	97.0 ± 8.1	95.8 ± 7.5	101.3 ± 8.7
Waist/Hip ratio	0.7 ± 0.06	0.8 ± 0.07	0.7 ± 0.05	0.8 ± 0.06	0.8 ± 0.07	0.8 ± 0.09
Systolic P. (mmHg)	120.5 ± 11	122.4 ± 14.2	120.3 ± 12.0	122.5 ± 15.9	120.8 ± 9.9	122.2 ± 10.9
DiastolicP. (mmHg)	82.0 ± 12.1	82.3 ± 7.2	81.1 ± 11.3	82.3 ± 6.05	83.2 ± 13.5	82.4 ± 9.3
Insulin (μU/ml)	19.0 ± 19.7	24.6 ± 25	18.0 ± 22.2	22.2 ± 19.4	20.4 ± 16.6	29.0 ± 33.4
Glycemia (mg/dL)	95.6 ± 23.7	99 ± 25.1	91.3 ± 22.7	100.2 ± 23.2	101.5 ± 24.7	96.8 ± 29.0
Total chol. (mg/dL)	187.1 ± 28.8	195 ± 35.6	182.1 ± 28.3	199.0 ± 35.5	194 ± 29.3	187.7 ± 35.9
HDL (mg/dL)	64.6 ± 18.8	62.0 ± 17.0	62.5 ± 17.2	62.8 ± 16.4	67.5 ± 21.4	60.6 ± 18.7
Triglycerides (mg/dL)	81.0 ± 43.8	102.7 ± 43	77.5 ± 40.4	102.8 ± 41.4	85.9 ± 49.9	102.6 ± 47.7
Fat mass (%)	27.8 ± 8.2	31.7 ± 7.0	27.6 ± 9.02	30.8 ± 6.6	28.1 ± 7.5	33.6 ± 7.6
Fat mass (kg)	17.6 ± 8.0	21.8 ± 10	17.6 ± 9.1	20.5 ± 8.9	17.6 ± 6.5	24.5 ± 11.9
GSL-TPAQ total score	32.2 ± 19.8	16.3 ± 13.9*	30.7 ± 20.4	17.8 ± 15.9*	34.4 ± 19.9	13.5 ± 9.3*
GSL-TPAQ strenuous intensity	8.0 ± 11.2	1.9 ± 6.0*	8.4 ± 11.5	2.6 ± 7.3	7.4 ± 11.3	0.69 ± 2.5*
GSL-TPAQ moderate intensity	14.4 ± 12.1	8.0 ± 10.1*	14.3 ± 11.6	6.3 ± 9.0*	14.5 ± 13.3	11.1 ± 11.6
GSL-TPAQ mild intensity	9.8 ± 8.4	5.8 ± 6.6*	8.2 ± 8.0	7 ± 6.8	12.0 ± 8.8	3.7 ± 5.8*

Mean ± SD values in *good* sleepers and *bad* sleepers.

*p < 0.05.

### Correlation analysis

Correlation analysis were performed to study the association between sleep quality, PA and anthropometric and metabolic parameters.

As regard sleep, we observed that a higher PSQI score, which corresponds to *bad sleep* quality, was significantly correlated with higher triglycerides levels (r_s_ = 0.29, *p* < 0.05). About PA, higher GSL-TPAQ score, which indicates greater PA levels, was significantly correlated with lower values of weight (r_s_ =  − 0.27, *p* < 0.05), BMI (r _s_ =  − 0.32, *p* < 0.05), waist circumference (r_s_ =  − 0.27, *p* < 0.05), hip circumference (r_s_ =  − 0.31, *p* < 0.01), insulin (r_s_ =  − 0.35, *p* < 0.01), triglycerides levels (r _s_ =  − 0.28, *p* < 0.05), fat mass in % (r_s_ =  − 0.36, *p* < 0.01), fat mass in kg (r_s_ =  − 0.31, *p* < 0.05), and PSQI score (r_s_ =  − 0.41, *p* < 0.01). Taking into account the single question of PA intensity, strenuous intensity PA was significantly correlated with lower values of weight (r_s_ =  − 0.33, *p* < 0.01), BMI (r_s_ =  − 0.32, *p* < 0.01), waist circumference (r_s_ =  − 0.41, *p* < 0.01), hip circumference (r_s_ =  − 0.34, *p* < 0.01), waist/hip ratio (r_s_ =  − 0.32, *p* < 0.05), systolic (r_s_ =  − 0.31, *p* < 0.01), diastolic blood pressure (r_s_ =  − 0.43, *p* < 0.01), triglycerides (r_s_ =  − 0.25, *p* < 0.05), fat mass in % (r_s_ =  − 0.47, *p* < 0.01) and fat mass in kg (r_s_ =  − 0.36, *p* < 0.01). Even though not significant, strenuous intensity PA showed a tendency to be correlated with lower PSQI score (r_s_ =  − 0.23, *p* = 0.07). Moderate intensity PA was significantly correlated with lower PSQI score (r_s_ =  − 0.34, *p* < 0.01). Mild intensity PA was significantly correlated with lower waist circumference (r_s_ =  − 0.28, *p* < 0.05), but showed no significant correlation with PSQI score (r_s_ =  − 0.15, *p* = 0.23).

### Binomial regression analysis

Table 3 shows the PR of being a *good sleeper* by considering the increasing tertiles of GSL-TPAQ total score. We observed that women in the higher tertile of GSL-TPAQ total score (≥ 27 METs/week) have a greater and significant PR of being a *good sleeper* compared to women in the lower tertile (≤ 11 METs/week). These results mean that women who practice strenuous PA for at least 3 times/week (or practice moderate PA 6 times/week) have about three times higher probability to have a better sleep quality. These results were consistent in both BRCA1 and BRCA2 even without any significant association.

**Table 3:** PRs of being a *good sleeper* according to tertiles of GSL-TPAQ total score.

	Total	BRCA 1	BRCA 2
**Tertiles GSL-TPAQ total score**	PR to be a *good sleeper* PR (95% CI)	PR to be a *good sleeper* PR (95% CI)	PR to be a *good sleeper* PR (95% CI)
1st Tertile GSL-TPAQ total score (0–11)	1	1	1
2nd Tertile GLS-TPAQ total score (12–26)	1.44 (0.54–3.82)	1.13 (0.33–3.90)	2.12 (0.28–15.9)
3rd Tertile GLS-TPAQ total score (≥ 27)	2.85 (1.25–6.52) (p = 0.01)*	2.34 (0.95–5.75) (p < 0.07)	5.16 (0.79–33.5) (p = 0.09)

PRs (95% CI) adjusted for age and BMI.

*p < 0.05.

We also estimated the PRs of being a *good sleeper* by unit of increase of MET/week (Table 4). In the whole population, as regards strenuous PA, every MET/week of increase was associated with a significant 10% increase of being a *good sleeper*. Similar results were obtained for moderate PA.

**Table 4:** PRs of being a *good sleeper* by unit of increase of MET/week for each question of PA intensity (strenuous, moderate, mild).

Variables	PR to be a *good sleeper* PR (95% CI)
GLS-TPAQ strenous intensity	1.10 (1.01–1.17) (*p* = 0.02)*
GSL-TPAQ moderate intensity	1.05 (1.0–1.11) (*p* = 0.04)*
GSL-TPAQ mild intensity	1.07 (1.0–1.15) (*p* = 0.06)

PRs ( 95% CI) adjusted for age and BMI.

*p < 0.05.

## Discussion

This cross-sectional analysis suggests a direct association between PA and sleep quality in 63 women carriers of *BRCA1/2* mutations. Women in the higher tertile of GSL-TPAQ total score (≥ 27 METs per week) have a greater and significant PR of being a *good sleeper* compared to women in the lower tertile (≤ 11 METs/week). The results were consistent also stratifying the population by BRCA mutation type. Furthermore, taking into account each single question of PA intensity, the PR of being a *good sleeper* by unit of increase of MET/week was higher and significant in women engaged in strenuous and moderate intensity PA. In contrast, mild intensity PA seemed not to significantly influence sleep behaviours.

To our knowledge, this is the first investigation assessing the potential influences of PA on sleep behaviour in this population. Previous studies in sporadic BC patients suggested the role of PA on the improvement of sleep behaviour (assessed both with actigraphy and questionnaires) and on the reduction of sleep deficiency at different stages of the BC pathology^26,41–43^.

Sleep quality in BC patients is essential for their quality of life. Indeed, BC diagnosis and treatments usually reflect on sleep as a long-term and side-effect symptom^44^. Quality of life usually decreases together with sleep after BC surgery and/or treatments, and their upgrading represents a challenging point for researchers^45,46^. The reasons explaining the bad sleep quality could be several: distress and anxiety of developing a very aggressive pathology or for the family's future; pain and hot flashes in consequent to surgery or treatments; fatigue that reduce the possibility to be sufficiently active^46,47^.

Sleep deficiency has been linked to increased inflammation and impaired immune response^48^. It also alters cellular signalling associated with mitochondrial respiratory function, insulin/IGF-I signalling^32^ and diminishes melatonin secretion, enhancing estrogen secretion^49–51^. Furthermore, sleep disorders decrease carbohydrate tolerance, alter leptin and cortisol levels^52,53^, thus modifying insulin sensitivity and favouring obesity and MS. For these mechanisms, sleep might represent one of the environmental factors involved in the modulation of BRCA penetrance. In this cross sectional analysis we decided not to report results by disease status because affected women showed a slightly worse sleep quality compared to unaffected, but without any significance result. Furthermore, the nature of the analysis and the small numbers of our study would not allow to draw conclusions about a potential effect of sleep on BRCA penetrance.

This cross-sectional analysis suggests that PA may be a tool for improving sleep quality in *BRCA1/2* women. Increasing PA levels and modifying daily habits in favour of a more active lifestyle has been among the leading research goals in the BC field also in order to improve sleep. The mechanisms on this linkage are not yet fully understood; however, studies from healthy subjects indicated that PA could exert its beneficial action on sleep through body temperature regularization, energy conservation, decrement in anxiety, stress, endocrine function, obesity and immune-inflammation response^54–58^. A regular and constant PA practice is recognized to improve body weight, BMI and other anthropometric parameters, insulin and IGF-I levels, adipokines balance, and sexual hormones secretion^59^. In our study, either total PA or strenuous intensity PA levels were correlated with lower weight, BMI, waist circumference, hip circumference, fat mass and insulin levels, suggesting a positive association also in *BRCA1/2* women.

The results of the present analysis should be seen in view of their limitations and strengths. Limitations may be attributable to the small sample size and to the use of questionnaires instead of objective assessment instruments for the all population (such as actigraphy). We are aware that is recommended to collect both, subjective and objective evaluations of sleep behavior and PA levels, but this was not foreseen by the MedDiet trial which had different aims^10^.

A further intrinsic limitation of this study is the cross-sectional design that does not allow to distinguish the cause from the effect. However, the intention of this paper was mainly to explore the sleep and PA data of the BRCA women who joined our MedDiet trial rather than to give an interpretation to the associations we found.

Despite the limitations, the preliminary results of the present analysis suggest new aspects to study in order to improve the quality of life in BRCA mutation carriers. The MedDiet results^10^ demonstrated that a dietary intervention in women carriers is feasible and effective in reducing metabolic and anthropometric parameters. Additional recommendations about PA for a more active lifestyle might be probably useful for the clinical management of this special group of women. These results represent therefore the seed for continuing the recruitment to broaden the sample and to evaluate the association between PA and sleep in a larger population of BRCA mutation carriers.

## Conclusions

The present study suggests an association between PA and sleep in women carriers of *BRCA1/2* mutations, highlighting that higher PA levels are associated with a better quality of sleep behaviour. In this perspective, future studies involving PA interventions are needed to improve sleep and quality of life in women predisposed to hereditary BC.
