# Author Correction: Physical activity and sleep behaviour in women carrying BRCA1/2 mutations

**DOI:** 10.1038/s41598-022-25014-7

**Published:** 2022-11-28

**Authors:** Letizia Galasso, Lucia Castelli, Eliana Roveda, Andreina Oliverio, Ivan Baldassari, Fabio Esposito, Antonino Mulè, Angela Montaruli, Patrizia Pasanisi, Eleonora Bruno

**Affiliations:** 1 Department of Biomedical Sciences for Health, University of Milan, Via G. Colombo 71, 20133 Milan, Italy; 2 IRCCS Istituto Ortopedico Galeazzi, Via R. Galeazzi 4, 20161 Milan, Italy; 3 Department of Research, Fondazione IRCCS Istituto Nazionale dei Tumori di Milano, Via G. Venezian 1, 20133 Milan, Italy

Correction to: *Scientific Reports* https://doi.org/10.1038/s41598-022-16687-1, published online 27 July 2022

The original version of this Article contained a spelling error in the name of the author Patrizia Pasanisi, which was incorrectly given as Pasanisi Patrizia.

The original Article has been corrected.

